# Baseline neutrophil-to- ratio combined with the change during treatment provides risk stratification for metastatic malignant melanoma patients treated with PD-1 inhibitors in a Chinese population

**DOI:** 10.3389/fonc.2023.1118301

**Published:** 2023-04-21

**Authors:** Chen Wang, Shengyan Liu, Xin Li, Kang Cui, Weijie Zhang, Yabing Du

**Affiliations:** ^1^ Department of Oncology, The First Affiliated Hospital of Zhengzhou University, Zhengzhou, China; ^2^ Department of Hepatopancreatobiliary Surgery, The First Affiliated Hospital of Zhengzhou University, Zhengzhou, China

**Keywords:** melanoma, PD-1 inhibitors, prognosis, baseline NLR, relative change of NLR

## Abstract

**Background:**

Previous studies have suggested that an elevated baseline neutrophil-to-lymphocyte ratio (BLNLR) and elevated relative change of NLR (ΔNLR%) is associated with worse outcomes in patients with a variety of cancers. This study aims to investigate the value of BLNLR and ΔNLR% before the third cycle of treatment on the prognosis of patients with metastatic malignant melanoma treated with PD-1 inhibitors.

**Methods:**

A total of 63 patients with metastatic malignant melanoma treated with PD-1 inhibitors in the First Affiliated Hospital of Zhengzhou University from January 2017 to December 2021 were retrospectively analyzed. BLNLR and ΔNLR% before the third cycle of treatment were collected. The Kaplan-Meier method was used to draw survival curves and Log-Rank test was used for survival analysis. Univariate and multivariate Cox regression analysis were used to analyze the relationship between BLNLR, ΔNLR% and clinical characteristics with progression-free survival (PFS) and overall survival (OS).

**Results:**

Univariate analysis showed that PFS and OS were associated with BLNLR, ΔNLR%, BMI and number of metastatic organs (*P* < 0.05). Multivariate analysis showed that BLNLR, ΔNLR%, BMI and number of metastatic organs were independent predictors of OS and BLNLR and ΔNLR% were independent predictors of PFS. Patients were divided into four groups according to BLNLR (<3, ≥3) and ΔNLR% (< 30%, ≥30%): low-BLNLR + low-ΔNLR% group, low-BLNLR + high-ΔNLR% group, high-BLNLR + low-ΔNLR% group, high-BLNLR + high-ΔNLR% group. The median OS was 20 months, 8 months, 9 months, 5 months and the median PFS was 8 months, 3 months, 2 months, 2 months, respectively.

**Conclusion:**

BLNLR combined with ΔNLR% can be used to predict the prognosis of PD-1 inhibitors in patients with metastatic malignant melanoma.

## 1 Introduction

Malignant melanoma is a highly aggressive malignant tumor, commonly found in the skin, digestive tract, genitals, nasal cavity and eyes (1). Early melanoma can be cured by surgical treatment, but the prognosis of advanced melanoma is poor. Before 2011, chemotherapy was the mainstay of treatment for advanced melanoma with a 5-year survival rate of less than 5% (2, 3). With the development of immune checkpoint inhibitors, the clinical outcomes of patients with advanced melanoma have been significantly improved and some patients can achieve long-term survival. The 5-year survival rate of patients with advanced melanoma treated with programmed cell death protein 1 (PD-1) inhibitors is about 40% (4–6). Nevertheless, the objective response rate of malignant melanoma treated with PD-1 inhibitors is low and more than half of patients cannot benefit from PD-1 inhibitors (4, 7, 8). In addition, PD-1 inhibitors are costly and some patients may experience serious immune-related adverse events. Therefore, it is crucial to identify appropriate prognostic markers and those patients who are most likely to respond to PD-1 inhibitors.

Although the drug development and clinical application of PD-1 inhibitors are progressing rapidly, only a few prognostic markers have been used in clinical practice such as programmed death ligand-1(PD-L1) expression, microsatellite instability (MSI) and tumor mutation burden (TMB) (9–13). These markers play an important role in predicting the efficacy of PD-1 inhibitors, but there are still some limitations due to the need for invasive biopsies to obtain tissue, intratumor heterogeneity in PD-L1 expression and high cost and so on (14). Peripheral blood-based markers such as neutrophil-to-lymphocyte ratio (NLR), platelet-to-lymphocyte ratio (PLR) and circulating tumor cells are easy to obtain and can be continuously evaluated, which has gained more and more attention.

Cancer-associated inflammation, recognized as one of the hallmarks of cancer, plays an important role in occurrence and development of tumors (15). NLR as a marker of inflammation in peripheral blood is cheap, readily available and easy-to-use. More and more evidence has shown that higher baseline NLR (BLNLR) is associated with poor prognosis of advanced melanoma patients receiving immunotherapy (16–19). In addition, BLNLR before immunotherapy and the relative change of NLR after immunotherapy (ΔNLR%) are also related to the prognosis of advanced patients in European and American populations mainly with cutaneous melanoma (20–22). However, the response of different melanoma subtypes to immunotherapy is highly heterogeneous. In the Chinese population, acral and mucosal melanoma are the main subtypes with a lower TMB, a higher proportion of chromosomal structural variation and a worse prognosis compared with cutaneous melanoma mainly occurs in European and American patients (23). Although current studies have found that BLNLR can predict the response to immunotherapy in the Chinese population (24), there is no report on the prognostic value of ΔNLR% in Chinese patients with advanced melanoma. The aim of this study is to investigate the prognostic value of BLNLR combined with ΔNLR% in patients with metastatic melanoma treated with PD-1 inhibitors in the Chinese population, to stratify the prognosis of patients and to attempt to provide reference for clinical decision making of metastatic melanoma patients in China.

## 2 Materials and methods

### 2.1 Patients selection

This study retrospectively analyzed the clinical data of patients with stage IV malignant melanoma treated with PD-1 inhibitors in the First Affiliated Hospital of Zhengzhou University from January 2017 to December 2021. Inclusion criteria: (1) patients with malignant melanoma confirmed by pathological examination; (2) malignant melanoma was classified as stage IV according to the 8th edition of TNM staging. (3) Eastern Cooperative Oncology Group Performance Status (ECOG PS) was 0-2. (4) at least 2 cycles of PD-1 inhibitors therapy. (5) complete clinical and hematological results and follow-up records. Exclusion criteria: (1) previous or concurrent history of other malignant tumors. (2) complicated with severe autoimmune diseases and hematological diseases. (3) long-term steroid therapy. (4) acute infection within 2 weeks before treatment. (5) no imaging examination was performed to evaluate the efficacy during treatment.

### 2.2 Clinicopathologic variables

The clinical data of patients within 1 week before PD-1 inhibitors were collected, including age, gender, body mass index (BMI), smoking history, drinking history, primary site, ECOG PS, tumor stage (TNM staging system, 8th edition), BRAF mutation status, number of metastatic organs, immunotherapy drugs, line of systemic therapy and NLR within 1 week before the first and third cycle of treatment. BLNLR was the ratio of neutrophil count to lymphocyte count before the first cycle of treatment. ΔNLR% was relative NLR change (calculated as % change), defined as follows: ΔNLR% = (NLR before the third cycle of treatment/BLNLR-1) *100.

### 2.3 Efficacy evaluation and follow-up

Follow-up data were obtained through regular visits to the hospital and telephone follow-up. Progression-free survival (PFS) was defined as the time from the start of PD-1 inhibitors to tumor progression or patient death. Overall survival (OS) was defined as the time from the start of PD-1 inhibitors to patient death or the end of follow-up. Efficacy was assessed by radiographic results according to response evaluation criteria in solid tumors version 1.1 (RECIST 1.1). The follow-up was terminated on August, 2022. The follow-up time ranged from 2 to 34 months with a median of 10 months

### 2.4 Statistical analysis

At present, the cut-off values of BLNLR and ΔNLR% in predicting the prognosis of patients with advanced melanoma have not been unified. BLNLR is often used as the cut-off value of 3, 4, or 5 (18, 19, 25) and ΔNLR% is often used as the cut-off value of 30% (21, 23). In this study, 3 and 30% were selected as the cut-off values for BLNLR and ΔNLR% respectively. Patients were divided into low-BLNLR group (<3) and high-BLNLR group (≥3); Low-ΔNLR% (<30%) group and high-ΔNLR% (≥30%) group; Low-BLNLR+ low-ΔNLR% group, low-BLNLR+ high-ΔNLR% group, high-BLNLR+low-ΔNLR% group, high-BLNLR+ high-ΔNLR% group. IBM SPSS Statistics 25.0 was used for statistical analysis and R4.1.0 was used to draw the survival curve. Measurement data with normal distribution were represented as mean ± SD and comparison between groups was conducted using the independent sample t test. Measurement data with skewed distribution were represented as M (Q1, Q3). Count data were described as absolute numbers (percentage) and comparison between groups was analyzed using the chi-square test, the continuity correction chi-square test or Fisher's exact test. Univariate and multivariate analyses were analyzed using the Cox hazard regression model. Variables with *P* <0.05 in univariate analysis were included in multivariate analysis. The Kaplan-Meier method was used to draw survival curves and Log-Rank test was used for survival analysis. All statistical tests were two-sided probability tests and *P* <0.05 was considered statistically significant.

## 3 Results

### 3.1 Baseline characteristics of the patients

A total of 63 patients with stage IV melanoma treated with PD-1 inhibitors were included in this study, including 32 males (50.8%) and 31 females (49.2%), aged 54.5 ± 12.4 years. The primary site was cutaneous in 13 cases (20.6%), mucosal in 19 cases (30.2%), acral in 13 cases (20.6%), ocular in 10 cases (15.9%) and unknown in 8 cases (12.7%). The ECOG PS of 57 (90.5%) patients was 0-1. There were 3 patients (4.8%) in stage M1a, 12 patients (19%) in stage M1b, 39 patients (61.9%) in stage M1c and 9 patients (14.3%) in stage M1d. 21 patients were treated with PD-1 inhibitors alone and the remaining patients were treated with PD-1 inhibitors combined with angiogenesis inhibitors, targeted therapy, chemotherapy or radiotherapy. 27 (42.9%) patients had multiple metastases and 36 (57.1%) patients had single organ metastasis. 27 (42.9%) received PD-1 inhibitor as first-line therapy and the remaining patients received second or more lines of treatment. The median PFS was 3 months [95% confidence interval (CI): 1.89-4.11 months] and the median OS was 11 months (95%CI: 8.57-13.43months). The 6-month, 1-year and 2-year OS rates were 71.4% (95%CI: 61.1%-83.5%), 41.3% (95%CI: 30.5%-55.8%) and 25.4% (95%CI: 15.1%-42.9%) respectively. The BLNLR, NLR before the third cycle of treatment and ΔNLR% were 2.411 (1.705, 3.422), 2.546 (1.749, 4.724) and 22.3% (-12.9%, 46.7%) respectively. Detailed clinical characteristics are provided in **Table 1**.

**Table 1:** Baseline characteristics of 63 patients with metastatic melanoma.

Characteristic	Overall (n=63)	BLNLR<3 (n=41)	BLNLR⩾3 (n=22)	*P* value	ΔNLR%<30% (n=35)	ΔNLR%≥30% (n=28)	*P* value
Age	54.5±12.4	52.3±11.8	58.6±12.5	0.053^b^	55.5±12.1	53.4±12.8	0.502
Gender				**0.043**			0.910
Male	32 (50.8)	17 (41.5)	15 (68.2)		18 (51.4)	14 (50.0)	
Female	31 (49.2)	24 (58.5)	7 (31.8)		17 (48.6)	14 (50.0)	
Alcohol-drinking history				0.461			0.498
No	58 (92.1)	39 (95.1)	19 (86.4)		31 (88.6)	27 (96.4)	
Yes	5 (7.9)	2 (4.9)	3 (13.6)		4 (11.4)	1 (3.6)	
Smoking history				0.097			0.892
No	49 (77.8)	35 (85.4)	14 (63.6)		27 (77.1)	22 (78.6)	
Yes	14 (22.2)	6 (14.6)	8 (36.4)		8 (22.9)	6 (21.4)	
Primary site				0.098^a^			**0.001^a^**
Cutaneous	13 (20.6)	10 (24.4)	3 (13.6)		3 (8.6)	10 (35.7)	
Mucosal	19 (30.2)	14 (34.1)	5 (22.7)		15 (42.9)	4 (14.3)	
Acral	13 (20.6)	5 (12.2)	8 (36.4)		11 (31.4)	2 (7.1)	
Ocular	10 (15.9)	5 (12.2)	5 (22.7)		3 (8.6)	7 (25.0)	
Primary site unknown	8 (12.7)	7 (17.1)	1 (4.5)		3 (8.6)	5 (17.9)	
ECOG PS				0.716			0.886
0-1	57 (90.5)	38 (92.7)	19 (86.4)		31 (88.6)	26 (92.9)	
2	6 (9.5)	3 (7.3)	3 (13.6)		4 (11.4)	2 (7.1)	
M stage				0.228^a^			0.453^a^
M1a	3 (4.8)	3 (7.3)	0 (0.0)		2 (5.7)	1 (3.6)	
M1b	12 (19.0)	10 (24.4)	2 (9.1)		9 (25.7)	3 (10.7)	
M1c	39 (61.9)	22 (53.7)	17 (77.3)		19 (54.3)	20 (71.4)	
M1d	9 (14.3)	6 (14.6)	3 (13.6)		5 (14.3)	4 (14.3)	
BMI(kg/m^2^)				0.259			0.337
<24	34 (54.0)	20 (48.8)	14 (63.6)		17 (48.6)	17 (60.7)	
≥24	29 (46.0)	21 (51.2)	8 (36.4)		18 (51.4)	11 (39.3)	
BRAF mutation				0.925^a^			0.873^a^
Yes	6 (9.5)	4 (9.8)	2 (9.1)		3 (8.6)	3 (10.7)	
No	32 (50.8)	20 (48.8)	12 (54.5)		17 (48.6)	15 (53.6)	
Unknown	25 (39.7)	17 (41.5)	8 (36.4)		15 (42.9)	10 (35.7)	
Combined with other therapy				0.455			0.473
No	21 (33.3)	15 (36.6)	6 (27.3)		13 (37.1)	8 (28.6)	
Yes	42 (66.7)	26 (63.4)	16 (72.7)		22 (62.9)	20 (71.4)	
Immunotherapy drugs				0.409			0.730
Toripalimab	35 (55.5)	21 (51.3)	14 (63.6)		21 (60.0)	14 (50.0)	
Pembrolizumab	18 (28.6)	14 (34.1)	4 (18.2)		9 (25.7)	9 (32.1)	
Camrelizumab	10 (15.9)	6 (14.6)	4 (18.2)		5 (14.3)	5 (17.9)	
Number of metastatic sites				**0.003**			1.000
1	36 (57.1)	29 (70.7)	7 (31.8)		20 (57.1)	16 (57.1)	
≥2	27 (42.9)	12 (29.3)	15 (68.2)		15 (42.9)	12 (42.9)	
Line of treatment				0.195			0.306
1	27 (42.9)	20 (48.8)	7 (31.8)		17 (48.6)	10 (35.7)	
≥2	36 (57.1)	21 (51.2)	15 (68.2)		18 (51.4)	18 (64.3)	

BMI, body mass index; ECOG PS, Eastern Cooperative Oncology Group performance status. a: Fisher's exact test. b: independent sample t-test. The bold values represent P<0.05.

### 3.2 The association between BLNLR, ΔNLR% and clinical characteristics of patients

In this study, statistically significant differences were observed in sex and number of metastatic organs between the low-BLNLR group and the high-BLNLR group (*P* <0.05). There were no significant differences in other characteristics (age, drinking history, smoking history, primary site, ECOG PS, M stage, BMI, BRAF mutation, combination therapy, immunotherapy drugs and line of systemic therapy between the two groups (*P* >0.05). There was a significant difference in the distribution of primary sites between the low-ΔNLR% group and the high-ΔNLR% group (*P* = 0.001) and no significant differences were observed in other characteristics (*P* >0.05) (**Table 1**).

### 3.3 High BLNLR and High ΔNLR% were associated with worse OS and PFS

Univariate Cox regression analysis showed that BMI<24kg/m^2^, number of metastatic organs ≥2, high BLNLR and high ΔNLR% were significantly associated with poor OS and PFS (*P* < 0.05) (**Table 2**).

**Table 2:** Univariate Cox regression model on overall survival and progression-free survival.

Characteristic	OS		PFS	
	HR (95%CI)	*P* value	HR (95%CI)	*P* value
Age	1.00 (0.98-1.03)	0.807	1.01 (0.98-1.03)	0.639
Gender				
Male	1		1	
Female	0.89 (0.49-1.61)	0.702	0.96 (0.57-1.62)	0.874
Alcohol-drinking history				
No	1		1	
Yes	0.90 (0.28-2.94)	0.867	1.35 (0.54-3.40)	0.520
Smoking history				
No	1		1	
Yes	1.59 (0.77-3.25)	0.208	1.28 (0.69-2.38)	0.442
Primary site				
Cutaneous	1		1	
Mucosal	1.12 (0.48-2.57)	0.799	1.19 (0.57-2.48)	0.641
Acral	0.90 (0.33-2.44)	0.832	0.90 (0.39-2.08)	0.801
Ocular	1.33 (0.49-3.58)	0.579	1.23 (0.50-3.02)	0.655
Primary site unknown	1.75 (0.61-5.03)	0.297	1.30 (0.53-3.18)	0.573
ECOG PS				
0-1	1		1	
2	1.27 (0.45-3.60)	0.649	1.04 (0.44-2.42)	0.935
M stage				
M1a	1		1	
M1b	1.19 (0.23-6.12)	0.833	1.65 (0.36-7.55)	0.520
M1c	2.42 (0.56-10.53)	0.239	2.36 (0.57-9.85)	0.239
M1d	3.02 (0.59-15.40)	0.184	1.69 (0.36-8.01)	0.506
BMI(kg/m^2^)				
<24	1		1	
≥24	0.36 (0.19-0.69)	**0.002**	0.52 (0.30-0.90)	**0.019**
BRAF mutation				
Yes	1		1	
No	1.54 (0.46-5.19)	0.482	2.96 (0.69-5.58)	0.208
Unknown	1.93 (0.57-6.59)	0.293	1.94 (0.67-5.66)	0.225
Combined with other therapy				
No	1		1	
Yes	1.30 (0.69-2.45)	0.422	1.08 (0.61-1.91)	0.800
Immunotherapy drugs				
Toripalimab	1		1	
Pembrolizumab	0.69 (0.33-1.44)	0.318	0.69 (0.37-1.28)	0.241
Camrelizumab	1.04 (0.48-2.28)	0.921	0.97 (0.47-2.10)	0.991
Number of metastatic sites				
1	1		1	
≥2	2.46 (1.30-4.65)	**0.006**	2.01 (1.18-3.44)	**0.011**
Line of treatment				
1	1		1	
≥2	1.64 (0.88-3.05)	0.120	1.52 (0.88-2.61)	0.130
BLNLR				
<3	1		1	
≥3	3.16 (1.65-6.05)	**0.001**	2.54 (1.44-4.47)	**0.001**
ΔNLR%				
<30%	1		1	
≥30%	2.60 (1.40-4.83)	**0.003**	1.88 (1.10-3.21)	**0.022**

BLNLR, neutrophil-to-lymphocyte ratio before the first cycle of PD-1 inhibitor; DNLR%, relative NLR change (calculated as % change) before the third cycle of PD-1 inhibitor; BMI, body mass index; ECOG PS, Eastern Cooperative Oncology Group performance status; OS, overall survival; PFS, progression-free survival. The bold values represent P<0.05.

**Figure 1:**
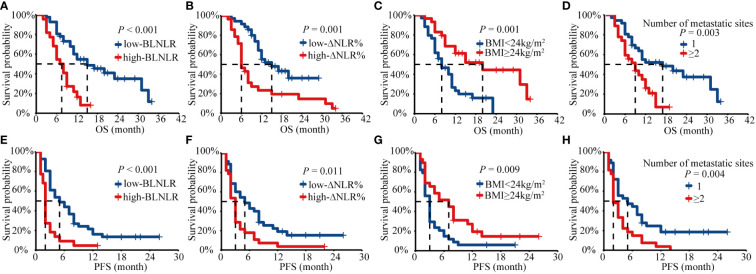
Kaplan-Meier analysis of OS and PFS for BLNLR, ΔNLR%, BMI, number of metastatic sites. **(A)** OS curve for BLNLR. **(B)** OS curve for ΔNLR%. **(C)** OS curve for BMI. **(D)** OS curve for number of metastatic sites. **(E)** PFS curve for BLNLR. **(F)** PFS curve for ΔNLR%. **(G)** PFS curve for BMI. **(H)** PFS curve for number of metastatic sites. BLNLR, neutrophil-to-lymphocyte ratio before the first cycle of PD-1 inhibitor; ΔNLR%, relative NLR change (calculated as % change) before the third cycle of PD-1 inhibitor; BMI, body mass index; OS, overall survival; PFS, progression-free survival.

The Kaplan-Meier survival curve showed that the median OS of the low-BLNLR group was 15 months (95%CI: 7.48-22.52 months) and only 7 months (95%CI: 4.70-9.30 months) in the high-BLNLR group (*P*<0.001) (**Figure 1A**). The median PFS of the low-BLNLR group was 5 months (95%CI: 2.31-7.70 months) and only 2 months (95%CI: 1.77-2.23 months) in the high-BLNLR group (*P*<0.001) (**Figure 1E**). The median OS of the low-ΔNLR% group was 15 months (95%CI: 7.75-22.25 months) and only 6 months (95%CI: 4.71-7.29 months) in the high-ΔNLR% group (*P*=0.001) (**Figure 1B**). The median PFS of the low-ΔNLR% group was 5 months (95%CI: 2.10-7.90 months) and only 3 months (95%CI: 2.50-3.50 months) in the high-ΔNLR% group (*P* = 0.011) (**Figure 1F**). The median OS of BMI≥24 kg/m^2^ group was 20 months (95%CI: 9.85-30.15 months) and only 8 months (95%CI: 4.82-11.18 months) in the BMI<24kg/m^2^ group (*P* = 0.001) (**Figure 1C**). The median PFS of BMI≥24kg/m^2^ group was 7 months (95%CI: 3.06-10.94 months) and only 3 months (95%CI: 2.42-3.58 months) in the BMI<24kg/m^2^ group (*P* = 0.009) (**Figure 1G**). The median OS of the single organ metastasis group was 17 months (95%CI: 8.63-25.37 months) and only 9 months (95%CI: 5.82-12.19 months) in the multiple organ metastasis group (*P*=0.003) (**Figure 1D**). The median PFS of the single organ metastasis group was 5 months (95%CI: 0.60-9.40 months) and only 2 months (95%CI: 1.36-2.64 months) in the multiple organ metastasis group (*P* = 0.004) (**Figure 1H**).

In a multivariate Cox regression model, BLNLR, ΔNLR%, BMI and number of metastatic organs were independent prognostic factors for OS (**Table 3**). The risk of mortality in the high-BLNLR group was 2.58 times higher than that in the low-BLNLR group (HR: 2.58, 95%CI: 1.21-5.52, *P* = 0.014). The risk of mortality in the high-ΔNLR% group was 3.76 times higher than that in the low-ΔNLR% group (HR: 3.76, 95%CI: 1.93-7.32, *P* < 0.001). BLNLR and ΔNLR% were independent prognostic factors for PFS (**Table 3**). The risk of disease progression in the high-BLNLR group was 2.20 times higher than that in the low-BLNLR group (HR: 2.20, 95%CI: 1.15-4.18, *P* = 0.017). The risk of disease progression in the high-ΔNLR% group was 2.44 times higher than that in low-ΔNLR% group (HR: 2.44, 95%CI: 1.37-4.32, *P* = 0.002) (**Table 3**).

**Table 3:** Multivariate Cox regression model on overall survival and progression-free survival.

Characteristic	OS		PFS	
	HR (95%CI)	*P* value	HR (95%CI)	*P* value
BLNLR				
<3	1		1	
≥3	2.58 (1.21-5.52)	**0.014**	2.20 (1.15-4.18)	**0.017**
ΔNLR%				
<30%	1		1	
≥30%	3.76 (1.93-7.32)	**<0.001**	2.44 (1.37-4.32)	**0.002**
BMI(kg/m^2^)				
<24	1		1	
≥24	0.33 (0.16-0.65)	**0.001**	0.58 (0.33-1.04)	0.066
Number of metastatic sites				
1	1		1	
≥2	2.32 (1.10-4.91)	**0.027**	1.65 (0.90-3.02)	0.103

BLNLR, neutrophil-to-lymphocyte ratio before the first cycle of PD-1 inhibitor; DNLR%, relative NLR change (calculated as % change) before the third cycle of PD-1 inhibitor; BMI, body mass index; OS, overall survival; PFS, progression-free survival. The bold values represent P<0.05.

### 3.4 BLNLR combined with ΔNLR% provides risk stratification

**Figure 2:**
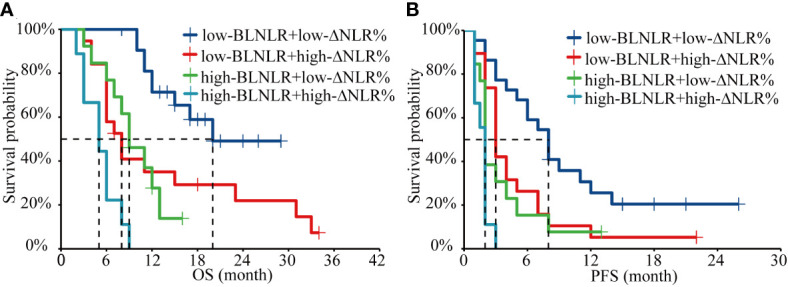
Kaplan-Meier analysis of OS and PFS for the combination of BLNLR and ΔNLR%. **(A)** OS curve. **(B)** PFS curve. BLNLR, neutrophil-to-lymphocyte ratio before the first cycle of PD-1 inhibitor; ΔNLR%, relative NLR change (calculated as % change) before the third cycle of PD-1 inhibitor; BMI, body mass index; OS, overall survival; PFS, progression-free survival.

In this study, both BLNLR and ΔNLR% were independent risk factors for OS and PFS and patients were stratified according to BLNLR and ΔNLR%. The patients in the low-BLNLR+ low-ΔNLR% group (n=22) had a good prognosis with a median OS of 20 months, which was significantly longer than the other three groups (all *P*<0.05, **Table 4**). The median OS of patients with one adverse prognostic feature was 8 months (95%CI: 5.34-10.66) and 9 months (95%CI: 5.70-12.30), respectively. The high-BLNLR+ high-ΔNLR% group (n=9) had the worst prognosis with a median OS of only 5 months (95%CI: 2.08-7.92) and all patients died within 10 months (**Figure 2A**). The median PFS of the low-BLNLR+ low-ΔNLR% group was 8 months (95%CI: 5.74-10.26), which was significantly longer than the other three groups (all *P*<0.05, **Table 4**; **Figure 2B**). The median PFS in the low-BLNLR+ high-ΔNLR% group, high-BLNLR+ low-ΔNLR% group and high-BLNLR+ high-ΔNLR% group were 3 months (95%CI: 2.30-3.70), 2 months (95%CI: 1.66-2.34) and 2 months (95%CI: 1.77-2.23) respectively (**Figure 2B**).

**Table 4:** Comparison between groups of Kaplan-Meier curves for low BLNLR+ low ΔNLR%, low BLNLR+ high ΔNLR%, high BLNLR+ low ΔNLR% and high BLNLR+ high ΔNLR%.

	*P* value	
	OS	PFS
Low-BLNLR+ low-ΔNLR% vs low-BLNLR+ high-ΔNLR%	**0.008**	**0.014**
Low-BLNLR+ low-ΔNLR% vs high-BLNLR+ low-ΔNLR%	**<0.001**	**0.005**
Low-BLNLR+ low-ΔNLR% vs high-BLNLR+ high-ΔNLR%	**<0.001**	**<0.001**
Low-BLNLR+ high-ΔNLR% vs high-BLNLR+ low-ΔNLR%	0.857	0.430
Low-BLNLR+ high-ΔNLR% vs high-BLNLR+ high-ΔNLR%	**0.008**	**0.001**
High-BLNLR+ low-ΔNLR% vs high-BLNLR+ high-ΔNLR%	**0.004**	**0.064**

BLNLR, neutrophil-to-lymphocyte ratio before the first cycle of PD-1 inhibitor; ΔNLR%, relative NLR change (calculated as % change) before the third cycle of PD-1 inhibitor; OS, overall survival; PFS, progression-free survival. The bold values represent P<0.05.

## 4 Discussion

Immunotherapy has become the main treatment for advanced melanoma, bringing significant survival benefits to patients. However, the therapeutic efficacy of immunotherapy in advanced melanoma is highly heterogeneous among different races and different individuals of the same race (25). A high BLNLR is associated with poor prognosis, but not all patients with low BLNLR respond well to immunotherapy. To address this clinical issue, we evaluated the prognostic value of BLNLR and ΔNLR% before third cycle of treatment and combined the two to stratify the prognosis of patients with metastatic malignant melanoma treated with PD-1 inhibitors.

Capone M et al. found that high BLNLR was an independent prognostic risk factor for OS and PFS in a retrospective study involving 97 patients with advanced melanoma who received immunotherapy (26). Qi et al. also confirmed that elevated BLNLR was associated with low immune response rate in the Chinese population (24). In a multicenter retrospective study of 224 patients with advanced melanoma receiving first-line immunotherapy, BLNLR was closely related to ECOG PS, number of metastatic sites and prognosis, and it was also found that ΔNLR% ≥30% during the first two cycles of treatment suggested an increased risk of death (20). Cassidy MR et al. dynamically examined NLR levels in melanoma patients receiving immunotherapy and similarly found that a 30% increase in NLR was an independent risk factor for prognosis (22). Consistent with the above studies, we found that patients with BLNLR≥3 and ΔNLR%≥30% had a significantly increased risk of death and disease progression. In addition, it was found that high-BLNLR was association with male and multiple organ metastases and high-NLR% was association with cutaneous, ocular unknown primary melanoma. These associations indirectly support that BLNLR and ΔNLR% reflect a comples and individualized interaction between both hosts and tumor factors.

NLR is a marker of systemic inflammatory response, but the mechanism of its effect on the prognosis of advanced melanoma immunotherapy is still unclear. Lymphocytes are the main effector cells of anti-tumor response and tumor-infiltrating lymphocytes can predict the efficacy of immunotherapy (27, 28). Several studies have shown that lymphopenia is associated with poor prognosis in a variety of tumors (29–31). Neutrophils reflect the inflammatory state of the host and are a marker of cancer (15). They are involved in the occurrence, proliferation and metastasis of tumors. Neutrophils can be recruited to inflammatory sites by chemokines and contribute to cancer initiation by DNA damage, angiogenesis and immunosuppression (32, 33). Neutrophil elastase (NE) produced by neutrophils can be endocytosed by tumor cells and then degrade insulin receptor substrate 1(IRS1) to free PI3K bound to IRS1, ultimately promoting tumor proliferation through the PI3K-AKT signal pathway (34). Neutrophils can also reduce the arginine in T cells by secreting arginase-1(ARG-1), thereby inhibiting the activation and proliferation of CD3 mediated T cells, ultimately leading to tumor proliferation (35). In addition, matrix metalloprotenase-9 (MMP-9) produced by neutrophils can promote the release of vascular endothelial growth factor (VEGF) to promote angiogenesis and participate in the process of tumor growth and metastasis (36). When it comes to tumor metastasis, neutrophils can do this by promoting cancer cell migration, intravasation and extravasation (33). Tumor-derived cathepsin G can induce cell migration and promote the entry of cancer cells into blood vessels. Neutrophil extracellular traps(NETs), a fibrous network composed of chromatin and proteins released from neutrophils, are able to capture circulating tumor cells and promote their dissemination at distant organs, leading to cancer metastasis (37). In addition, NE and MMP-9 can cleat membrane protein to induce the proliferation of dormant cancer cells in metastatic sites (38). A high NLR level may indicate an enhanced inflammatory response in the tumor microenvironment and a weakened lymphocyte-mediated anti-tumor response, thus reflecting the prognostic characteristics of melanoma patients.

The important problem of immunotherapy is the low response rate. In the Chinese population, the objective response rate of PD-1 inhibitors in patients with advanced melanoma is 15%-18.2% (39–43). Therefore, it is very important to find prognostic markers for immunotherapy in Chinese patients with malignant melanoma. BLNLR can predict the outcome of PD-1 inhibitors treatment in Chinese melanoma patients (24). However, the treatment of cancer is a dynamic process, so the dynamic change of NLR is more likely to better respond to the prognosis of PD-1 inhibitors treatment of melanoma patients. In this study, we found that the combination of BLNLR and ΔNLR% before the third cycle of treatment could enhance the predictive ability and better distinguish the prognosis of patients with melanoma under PD-1 inhibitors treatment. Melanoma patients with BLNLR <3 and ΔNLR% < 30% (34.9%) had a median OS of 20 months, which was significantly longer than the other three groups and were more likely to benefit from PD-1 inhibitors. In contrast, patients with BLNLR≥3 and ΔNLR%≥30% (14.3%) had extremely poor survival, with a median OS of only 5 months and all died within 10 months. In addition, it should be emphasized that the time point before the third cycle of treatment was chosen in this study, which was consistent with the time point when the first imaging examination was performed after treatment to assess the efficacy. If imaging examination before the third cycle treatment indicated stable or slight progression and the patient had low BLNLR and low ΔNLR%, it could be considered safe to continue this treatment. On the contrary, if the imaging examination before the third cycle of treatment indicates that the disease is slightly progressive but does not reach the progressive standard and the patient belongs to the high BLNLR and high ΔNLR% groups, doctors and patients should be cautious about the prognosis of such patients and there is a certain risk of disease progress in continuing PD-1 inhibitors. It has come to our knowledge that this is the first study to demonstrate in Chinese population that the combination of BLNLR and ΔNLR% enables prediction of prognosis in melanoma patients receiving PD-1 inhibitors. These results provide a strong basis for the combined use of BLNLR and ΔNLR% as prognostic markers to optimize patient benefit.

This study is a small sample and single-center retrospective study with potential selection bias and confounding factors. Due to the rarity of melanoma in the Chinese population, the number of patients included in this study was only 63. Subgroup analysis was not performed to further explore the effect of BLNLR and ΔNLR% on prognosis under different treatment methods. Based on the above limitations, further multi-center and large-sample prospective studies are needed to verify the conclusions of this study.

BLNLR combined with ΔNLR% can enhance the prognostic ability of metastatic melanoma patients treated with PD-1 inhibitors. Patients with low BLNLR and low ΔNLR% before the third cycle of treatment are more likely to benefit from PD-1 inhibitors. On the contrary, high BLNLR and high ΔNLR% is association with a very poor prognosis, which combined with other clinically relevant details such as imaging examination may be the reason for the change of treatment in this subgroup of patients. In conclusion, BLNLR combined with ΔNLR% can simply, economically and dynamically reflect the prognosis of metastatic melanoma, assisting clinicians in selecting treatment strategies to a certain extent.

## Data availability statement

The raw data supporting the conclusions of this article will be made available by the authors, without undue reservation.

## Ethics statement

The studies involving human participants were reviewed and approved by Ethics Committee of The First Affiliated Hospital of Zhengzhou University. Written informed consent for participation was not required for this study in accordance with the national legislation and the institutional requirements.

## Author contributions

CW, WZ, and YD performed the literature search and designed the study. CW, SL, XL, and KC contributed to the acquisition, analysis, and interpretation of the data. CW and SL wrote the original draft of the manuscript. WZ and YD edited the manuscript. All authors contributed to the article and approved the submitted version.
